# Mining data from legacy taxonomic literature and application for sampling spiders of the *Teutamus* group (Araneae; Liocranidae) in Southeast Asia

**DOI:** 10.1038/s41598-020-72549-8

**Published:** 2020-09-25

**Authors:** F. Andres Rivera-Quiroz, Booppa Petcharad, Jeremy A. Miller

**Affiliations:** 1 Department of Terrestrial Zoology, Understanding Evolution group, Naturalis Biodiversity Center, Darwinweg 2, 2333CR Leiden, The Netherlands; 2 Institute of Biology Leiden (IBL), Leiden University, Sylviusweg 72, 2333BE Leiden, The Netherlands; 3 Faculty of Science and Technology, Thammasat University, Rangsit, 12121 Pathum Thani Thailand; 4 Plazi, Zinggstrasse 16, CH 3007 Bern, Switzerland

**Keywords:** Biodiversity, Biogeography, Conservation biology

## Abstract

Taxonomic literature contains information about virtually ever known species on Earth. In many cases, all that is known about a taxon is contained in this kind of literature, particularly for the most diverse and understudied groups. Taxonomic publications in the aggregate have documented a vast amount of specimen data. Among other things, these data constitute evidence of the existence of a particular taxon within a spatial and temporal context. When knowledge about a particular taxonomic group is rudimentary, investigators motivated to contribute new knowledge can use legacy records to guide them in their search for new specimens in the field. However, these legacy data are in the form of unstructured text, making it difficult to extract and analyze without a human interpreter. Here, we used a combination of semi-automatic tools to extract and categorize specimen data from taxonomic literature of one family of ground spiders (Liocranidae). We tested the application of these data on fieldwork optimization, using the relative abundance of adult specimens reported in literature as a proxy to find the best times and places for collecting the species (*Teutamus politus*) and its relatives (*Teutamus* group, TG) within Southeast Asia. Based on these analyses we decided to collect in three provinces in Thailand during the months of June and August. With our approach, we were able to collect more specimens of *T. politus* (188 specimens, 95 adults) than all the previous records in literature combined (102 specimens). Our approach was also effective for sampling other representatives of the TG, yielding at least one representative of every TG genus previously reported for Thailand. In total, our samples contributed 231 specimens (134 adults) to the 351 specimens previously reported in the literature for this country. Our results exemplify one application of mined literature data that allows investigators to more efficiently allocate effort and resources for the study of neglected, endangered, or interesting taxa and geographic areas. Furthermore, the integrative workflow demonstrated here shares specimen data with global online resources like Plazi and GBIF, meaning that others can freely reuse these data and contribute to them in the future. The contributions of the present study represent an increase of more than 35% on the taxonomic coverage of the TG in GBIF based on the number of species. Also, our extracted data represents 72% of the occurrences now available through GBIF for the TG and more than 85% of occurrences of *T. politus*. Taxonomic literature is a key source of undigitized biodiversity data for taxonomic groups that are underrepresented in the current biodiversity data sphere. Mobilizing these data is key to understanding and protecting some of the less well-known domains of biodiversity.

## Introduction

In the aggregate, traditional taxonomic publications can be thought of as a repository that has accumulated vast amounts of biological data linked to specific taxonomic names. These units of taxonomic knowledge, information linked to a name within a publication, are known as taxonomic treatments^1–3^. This makes taxonomic literature not only crucial for the exchange and growth of biodiversity knowledge, but also capable of being used to detect and understand larger biodiversity patterns with historical perspective.

In recent years, great efforts have gone into the digitization of legacy taxonomic literature^4–6^. This combined with digital publications have greatly improved access to taxonomic literature. Nevertheless, although easy to share, PDF publications still have most biodiversity data embedded in strings of text making them less dynamic and difficult or impossible to read and analyze without a human interpreter^7^. This difficulty to access and use core specimen data is what we define as PDF prison^8^. Recently developed tools allow text in PDF documents to be interpreted and categorized in XML format (mark-up) allowing information to be mobilized, aggregated and reanalyzed^9–12^. Plazi Treatment Bank^8,13,14^, is a project dedicated to creating a comprehensive compendium of taxonomic and biological data extracted from primary literature^15^. This platform permits mined treatment data to be accessed, queried, compared, and reused in a customized way. The strategy for data extraction can be prospective: where journals generate new data in XML format that can be uploaded directly to repositories (as has been implemented by Zookeys^2^ and EJT^8,13^). or retrospective: where data is mined from legacy taxonomic literature^3,11–13^ through a process called semantic enhancement^9,13^. This retrospective approach is more complicated and time consuming since the semi-automatic process of text recognition and tagging needs to be checked by a human operator^3,15^. However, it can provide useful information by extracting, integrating and using biodiversity data contained in the hundreds of years of accumulated taxonomic literature. Data integration is achieved by linking records from Plazi treatment bank to the Global Biodiversity Information Facility (GBIF)^8,16^ where they are aggregated with other type of records, mainly natural history institution specimen collections and observation data based on GBIF’s taxonomic backbone^17^.

Here we combined several of these cybertaxonomic tools to test the data extraction process and its potential application on the design and planning of an expedition to collect fresh material in the field. We targeted the ground spider *Teutamus politus* Thorell 1,890 and its relatives from the so called *Teutamus* group (TG) (Araneae, Liocranidae)^18^. This group of spiders is mostly distributed in Southeast Asia^19–23^ and is composed of seven genera: *Jacaena*, *Koppe*, *Oedignatha*, *Sesieutes*, *Sphingius*, *Sudharmia* and *Teutamus*^18^. These spiders have been cataloged in the family Liocranidae; however, their phylogenetic relationships, biology and evolution are still poorly understood^18,24^. Therefore, collection of fresh specimens of the target taxa was necessary for building a molecular phylogeny of the TG. The species *T. politus*, besides being the type species of the genus *Teutamus*, is an example of the extremely rare phenomenon of directional genital asymmetry^25^. For this reason, the collection of live adult specimens was crucial to study, document, and test the behavioral implications of their abnormal genital morphology.

Our study aimed to highlight the importance of making biodiversity data contained within taxonomic treatments accessible and reusable in accordance with the FAIR data principles^26^. This approach can help bridge gaps and focus efforts in the study of particularly interesting taxa or geographic regions. The usability of taxonomic literature data, potential applications, and its limitations and biases are discussed.

## Results

### Literature data analysis

**Figure 1:**
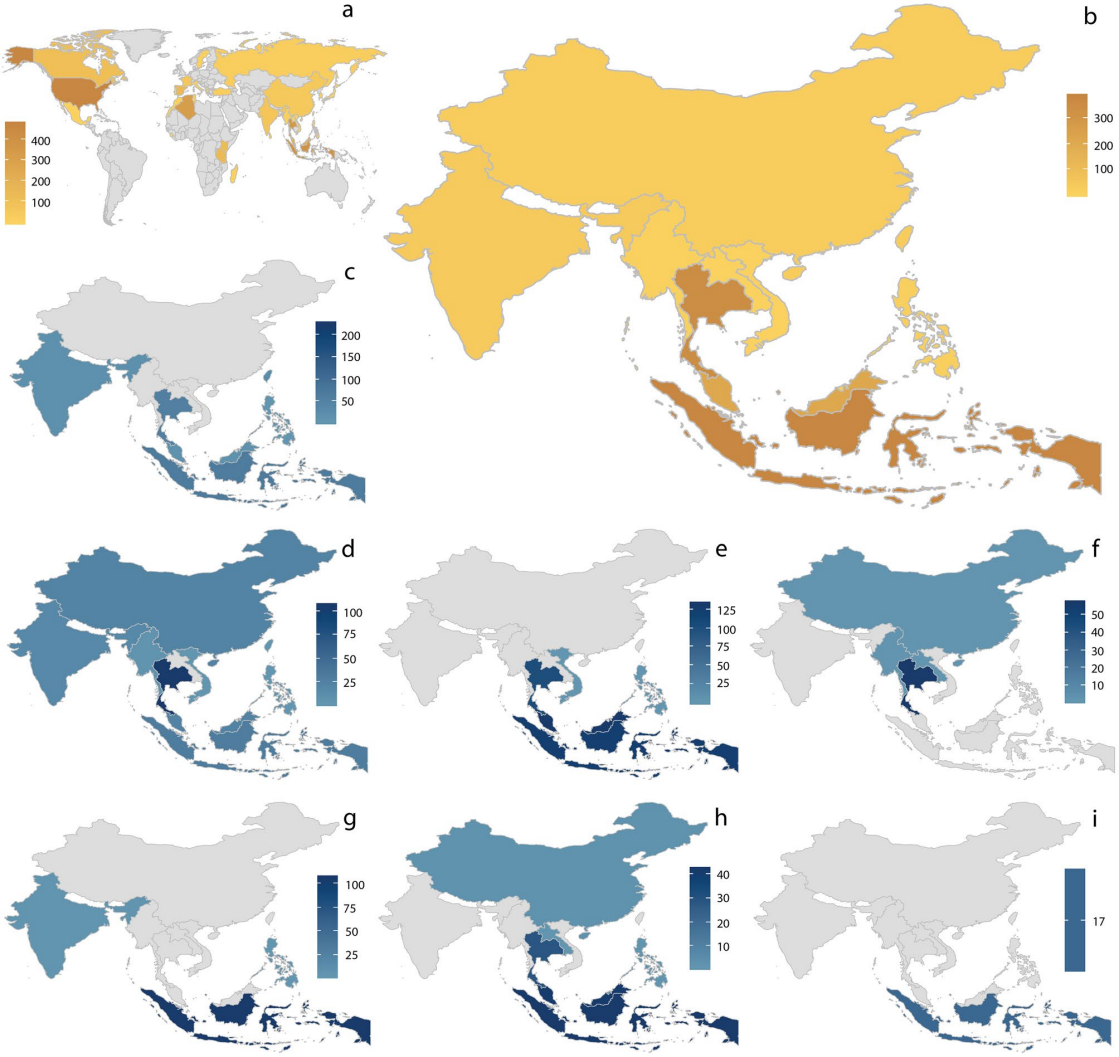
Maps of liocranid spiders distribution based on geographic data extracted from taxonomic literature using Plazi’s retrospective workflow (see Supplementary Table 1 for the whole set of documents used). Maps generated in RStudio^28–30^. (**a**) Family: Liocranidae worldwide. (**b**) Family Liocranidae in Southeast Asia (SEA). (**c**) Genus: *Oedignatha.* (**d**) *Sphingius*. (**e**) *Teutamus*. (**f**) *Jacaena*. (**g**) *Koppe*. (**h**) *Sesieutes*. (**i**) *Sudaharmia*. Brown shades represent family distribution and blue shades represent genus distributions. Color intensity corresponds to numbers of specimens per country.

**Figure 2:**
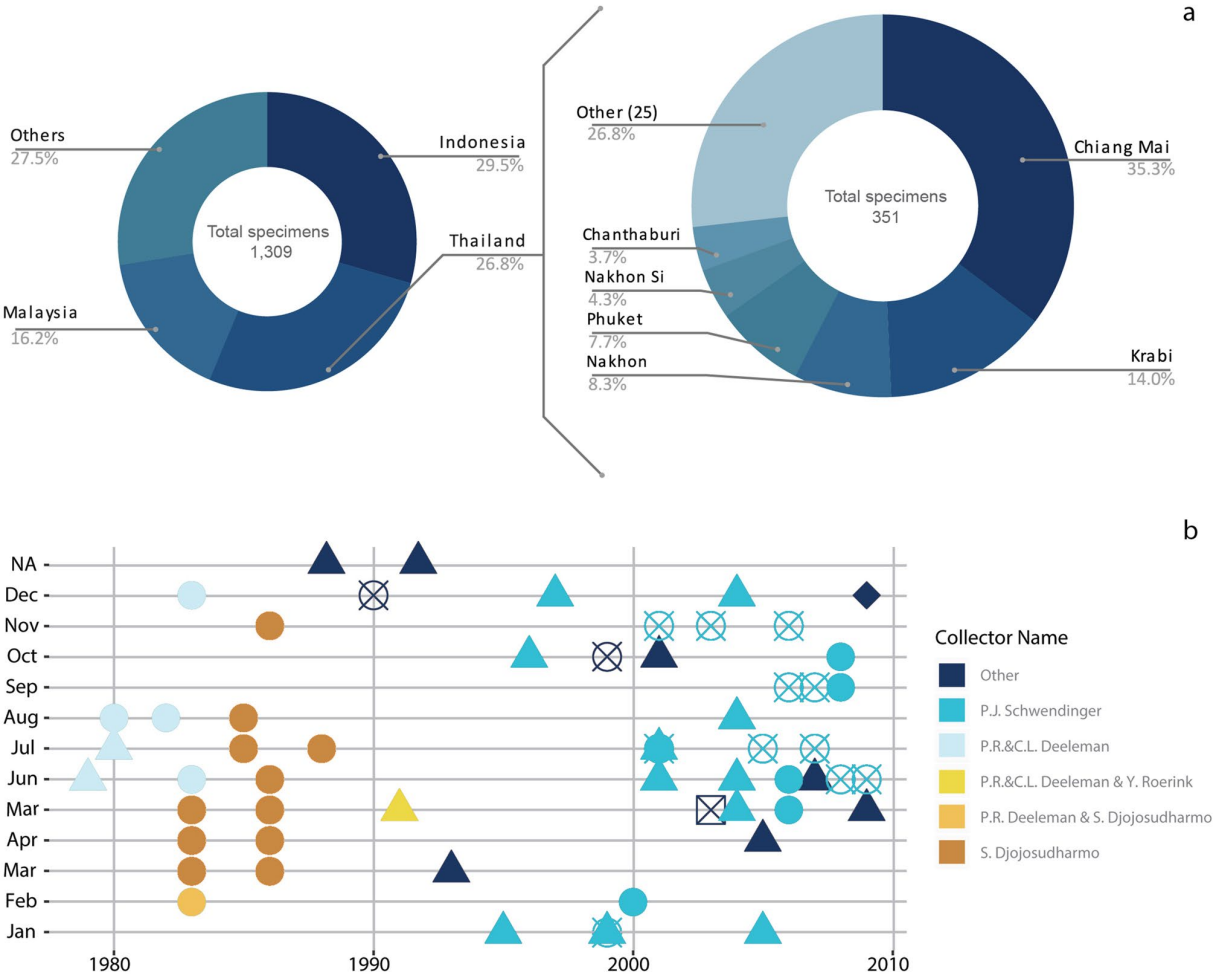
Distribution of the *Teutamus* group in Southeast Asia according to taxonomic literature (based on data extracted from 23 studies^19–23,31–48^ using Plazi’s retrospective workflow). (**a**) Proportion of specimens reported per country, with detail of provinces in Thailand. (**b**) Temporal and spatial distribution of collections for the past 40 years. ● = Indonesia, ▲ = Malaysia, ⦻ = Thailand, ◆ = Philippines, ⊠ = Vietnam.

Data extracted from 55 analyzed publications represent in total 23 genera and ca. 160 species of the family Liocranidae with ca. 3,000 specimens collected worldwide (Fig. 1a). A visual summary of the data extraction process and data display in Plazi’s Treatment Bank and GBIF can be found in Supplementary Figure 1. These include treatments of all currently valid genera and 90 species of the TG based on 1,309 specimens; out of 137 currently valid species^27^. The TG was mostly distributed in East and Southeast Asia (Fig. 1b) with the exception of two species of the genus *Oedignatha* found in the Seychelles. Within SEA, six genera of the TG have a broad distribution being reported from India and the southern region of mainland Asia to the Malay Archipelago (Fig. 1c–e,g–h). Two exceptions are *Jacaena* that has not been reported south of Thailand (Fig. 1f) and *Sudaharmia* that has only been reported within Indonesia (Fig. 1i). Indonesia (Six genera, 386 specimens), Thailand (Five, 351) and Malaysia (Four, 212) were the countries with a highest richness and abundance of TG spiders accounting for 72.5% of all the TG records (Fig. 2a). Thailand was the country that combined most occurrences of the TG genera and *T. politus* having 66% of all the known specimens of this species reported in literature. Within Thailand, the best sampled province is Chiang Mai accounting for 35% of all the TG specimen records for the country. Other relatively well known provinces were Krabi, Nakhon Ratchasima and Phuket, adding up to 30% of the country records (Fig. 2a). Chiang Mai had reports of four TG genera and 11 species, Krabi and Phuket had relatively less representation of the TG; however, these two provinces had 66 of the 68 specimens of *T. politus* recorded for the country.

The majority of species treatments that we semantically enhanced contained collecting dates that allowed us to plot temporal distribution of the group within Thailand. Most specimens were collected between 1980 and 2009. These dates together with collecting locations allowed us to plot the known temporal and geographic distribution of our target taxon (Fig. 2b). For instance, most collecting is concentrated between May and December, with February and March being the least represented months. Similarly, Indonesia, Malaysia and Thailand are the best sampled countries in Southeast Asia. From an historical perspective, Indonesia was clearly the most sampled area during the 80 s and Malaysia during the 90 s, with more heterogeneous and international records appearing during the 2000s.

**Figure 3:**
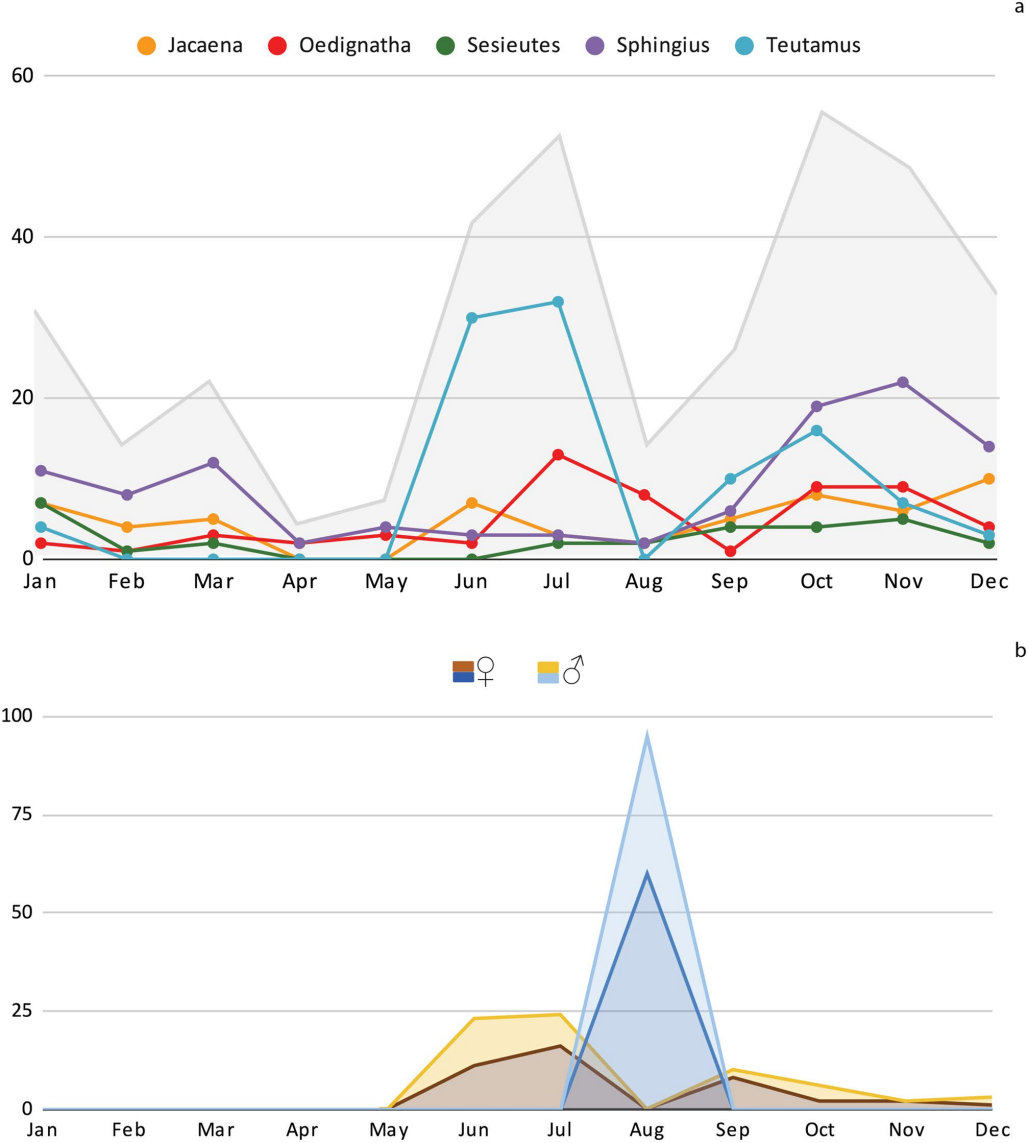
Seasonal distribution of adult specimens of the *Teutamus* group in Thailand based on data extracted from 2 studies^19,21^ using Plazi’s retrospective workflow. (**a**) Grey area indicates total number of specimens; lines detail richness per genus in literature. (**b**) Relative abundances of males and females of *Teutamus politus.* Brown shades indicate specimens in literature; blue shades indicate specimens in our study.

Total monthly abundances suggest that adults of the TG are mostly found in between June and July, and October to January (Fig. 3a). A more detailed visualization at genus level shows that most TG genera have similar seasonal variations, with the exception of *Teutamus* that is most common between June and July (Fig. 3a). The species *T. politus* has adults reported mostly between June and July, and some specimens from September to December but none have been recorded between January and May (Fig. 3b).

### Fieldwork

Our sampling produced 134 adult liocranid specimens from the following genera: *Jacaena* (3), *Oedignatha* (32), *Sesieutes* (3), *Sphingius* (1), *Teutamus* (95) (Table 1). Some juvenile specimens of *Oedignatha* and *Teutamus* could be matched to adults in the same sample and assigned to the same species adding up to a total of 229 identified specimens of the Liocranidae. We found four species of the TG in Chiang Mai: *Jacaena lunulata*, *Oedignatha barbata*, *O. jocquei*, and *Sphingius* cf. *vivax*; three species in Phuket: *O. spadix*, *Sesieutes* cf. *minuatus*, and *Teutamus politus*; and two species in Krabi: *O.* sp. and *T. politus.* Most of them were represented by males and females with the exception of *J. lunulata* and *S.* cf. *vivax*, where only males were found. These two, along with *O. barbata* and *O.* sp., were the rarest species having three or fewer individuals in our sample. The most abundant species were *O. spadix* and *T. politus* with 21 and 95 adults respectively.

**Table 1:** Records of *Teutaumus* group (TG) species from three Thai provinces.

Province	Species	Spp. in literature			Spp. July–August			Spp. in our study		
		♂	♀	Total	♂	♀	Total	♂	♀	Total
Chiang Mai	*Jacaena angoonae*	–	4	**4**	–	–	**–**	–	–	**–**
	*Jacaena lunulata*	8	5	**13**	–	–	**–**	3	–	**3**
	*Jacaena mihun*	3	3	**6**	–	–	**–**	–	–	**–**
	*Jacaena schwendingeri*	3	9	**12**	–	3	**3**	–	–	**–**
	*Oedignatha barbata*	6	5	**11**	2	2	**4**	1	1	**2**
	*Oedignatha jocquei*	8	15	**23**	6	9	**15**	1	6	**7**
	*Sesieutes zhui*	5	4	**9**	–	–	**–**	–	–	**–**
	*Sphingius gothicus*	16	6	**22**	–	–	**–**	–	–	**–**
	*Sphingius penicillus*	17	3	**20**	–	–	**–**	–	–	**–**
	Sphingius vivax*	–	–	**–**	–	–	**–**	1	–	**1**
Krabi	*Oedignatha* sp.*	–	–	**–**	–	–	**–**	1	1	**2**
	*Sesieutes aberrans*	2	–	**2**	2	–	**2**	–	–	**–**
	*Sphingius punctatus*	–	1	**1**	–	–	**–**	–	–	**–**
	***Teutamus politus***	20	19	**39**	1	–	**1**	5	14	**19**
	*Teutamus rama*	4	3	**7**	–	–	**–**	–	–	**–**
Phuket	Oedignatha spadix*	–	–	**–**	–	–	**–**	6	15	**21**
	*Sesieutes* cf. minuatus*	–	–	**–**	–	–	**–**	2	1	**3**
	***Teutamus politus***	8	19	**27**	7	16	**23**	30	46	**76**
Total specimens		100	96	**196**	18	30	**48**	50	84	**134**

Total records from taxonomic literature (Spp. in literature) vs. Literature records from June–August (Spp. July–August) vs. our field samples (Spp. in our study). *indicates new geographic distribution for the species.

## Discussion

### Literature data analysis

Detecting and understanding biodiversity patterns require large amounts of high quality data. In recent years global databased like GBIF and Plazi have set standards for collection, curation and dissemination of these biological data. GBIF, the largest biodiversity data repository, has aggregated digitized specimen records from many of the world’s most important biodiversity collections institutions. In addition, records from observation networks such as iNaturalist are aggregated on GBIF. However, legacy taxonomic literature as a source of biodiversity data has remained relatively unexplored until recent years. Taxonomic literature holds a vast amount of high-quality biodiversity data^12,49,50^. Like data from institutional collections and unlike data from observations networks, these data typically point to specimen objects archived in a natural history institution. Such records have the potential to be re-evaluated in a way that records from observation networks cannot be. It is worth noting that many specimens cited in the taxonomic literature, although archived in a natural history collection, are not necessarily among the institutional collections data shared with GBIF.

Data extraction from taxonomic literature can proceed along two major pathways: (1) prospective, where data is mobilized and shared with GBIF as part of the routine publication process, as has been implemented some journals like EJT^13^ and ZooKeys^2,8^ and some revisionary studies^51^; and (2) retrospective, where data is mined from legacy taxonomic data^11,12^. This retrospective approach was tested in our study by semantically enhancing records from more than 50 legacy taxonomic documents. From these sources, ca. 3,000 specimens of the family Liocranidae were structured and mobilized, including more than 1,300 records from about 100 treatments of TG taxa (Supplementary Table 1). These data included relevant biodiversity information, such as geographical distribution, date of collection, sex, and number of specimens.

Although the data contained in taxonomical treatments has been curated by specialists and is highly dependable, it is not free from error and methodological bias. Meyer, Weigelt, and Kreft^52^, in their study of land plant data available on GBIF, documented data biases in two major groups: *coverage* (geographical and temporal documentation gaps) and *uncertainty* (accuracy or credibility). Another bias observed in GBIF, as well as biodiversity studies and funding in general, is related to the taxonomic coverage and over representation of some groups like birds and plants and under representation of megadiverse groups like insects and arachnids^53–56^ (Supplementary Table 2; see also Data Aggregation, below).

In our analysis we did not find clear cases of *uncertainty* bias with the exception of the absence of geographical coordinates that made some of the occurrences spatially ambiguous. However, geographical and temporal *coverage* bias was observed. Scientists do not sample randomly or evenly from the whole world; therefore, it should be expected that some areas and times are studied more than others. This makes it difficult to distinguish seasonal changes in abundance from uneven sampling effort at different times of the year. Nevertheless, existing records at least indicate the time of year when specimens have been found in the past, and might therefore be found again. Overall, records of TG taxa were not evenly spread throughout the year. For example, zero specimens of *T. politus* are recorded for the month of August, suggesting that this might not be best time of year to search for this species in Thailand (Figs. 2, 3). Although we had planned our sampling during the highest abundance peak (June–July; Fig. 3b), logistic constrains forced us to carry our sampling one month later. Nevertheless, we found a total of 188 specimens of this species during our collection, of which 95 were adults. Our results give evidence of the presence of these taxa during this time of the year, suggesting that the variation observed in legacy records is most probably due to temporal *coverage* bias and must be interpreted with care.

Another temporal *coverage* bias was observed when assessing specimen contributions per collector (Fig. 2b). We found P.J. Schwendinger to be the collector with most specimens contributed to the TG^19–23^; between 1983 and 2009 he collected 231 TG specimens in Thailand. However, most of his specimens, presumably, due to logistics, were reported around June and July, and December. Therefore, temporal distribution patterns, as observed in literature-extracted data (Figs. 2 and 3), could be an artifact of sampling bias and not necessarily reflect real seasonal variation of the taxa.

Even taking into account these methodological biases, we consider specimen records in taxonomic literature to be among the best curated evidence of presence and, to some extent, relative abundances; and for many understudied and megadiverse taxa, this is the only source of specimen records available. Identifying and understanding data biases can help to identify temporal and spatial gaps were further sampling effort is needed.

### Fieldwork

Data extracted from taxonomic literature on the family Liocranidae were used to create detailed profiles for the TG. These helped us to plan a collection that specifically targeted the re-collection of these taxa. Our analysis showed that within Southeast Asia, three provinces in Thailand, Chiang Mai, Phuket and Krabi were the best choice for targeting *T. politus* and its relatives.

This selection of times and places, in combination with specific methods for collecting ground spiders showed a high efficiency for sampling the TG. Our one-month expedition captured 134 adult spiders of the TG (Table 1) representing all TG genera previously reported for Thailand and six out of seven liocranid genera reported for this country (only missing *Paratus* Simon, 1898). In total, 351 adults of the TG had been reported from Thailand^19–23,41^; from these, ca. 200 had been reported in the same provinces we sampled (Chiang Mai, Krabi and Phuket) (Table 1). When comparing only the collections reported for the same months where we sample, we can observe that our approach was much more efficient, collecting 134 adults vs. 48 in literature. We collected a total of nine TG species vs. 14 reported from the same provinces and six reported from the same provinces and times. From these, *Teutamus politus* was the most abundant species in both literature and our study with 66 and 95 adults respectively (Fig. 3b).We collect more specimens of this species (188) than all the previous records in literature combined (102 specimens)^19,21^. *Oedignatha spadix* was the second most abundant in our study with 21 adult specimens; *Oedignatha spadix* is previously known only from Indonesia^19^.

### Data aggregation

The interoperable network of Plazi allows the extracted data to be automatically shared with other biodiversity databases like GBIF. This allows taxonomic literature data to be analyzed together with data from Natural History collections and observation networks. Many studies have explored the limits and capabilities of GBIF data for setting conservation priorities^57–60^, modeling^57,61,62^, aggregation of different kinds of data and its biases^52,56,59,60,63,64^, among others. The major GBIF data domains (institutional collections databases, observation networks, taxonomic literature, and, in some cases, DNA sequence databases), each have their particular biases, but taken together are complementary enough to serve as a basis for building more complete biodiversity knowledge. In the case of the *Teutamus* group, virtually all records in GBIF were originated from digitized collection data with only five records contributed through human observation and one through iBOL^65^. Even in groups where other sources of data are not available, digitized collection data can give important insights on aspects like the group taxonomy and distributions. Two studies in the Amazonia highlight the importance of collection-based data, by aggregating museum specimen data of several unrelated taxa collected in Amazonia comparing their richness, distribution and endemism^66,67^. This approach allowed them to identify undersampling bias taxonomically and spatially, and map priority areas for conservation based on biodiversity data. They also observed that even when individual datasets might be imperfect, the aggregation of different approaches and sources can help to better assess and allocate conservation efforts.

In our study, the addition of records from the taxonomic literature, aggregated with complementary data from other sources available on GBIF, improved the taxonomic, geographic, and seasonal coverage of TG taxa (Table 2), giving us an improved picture of their overall biodiversity pattern. Semantic enhancement of taxonomic literature cannot compete in volume against the millions of records sourced from natural history collections databases and especially observation networks. But records from taxonomic literature may be the only source of data available for the vast portion of biodiversity about which we know very little. In other words, observation network records tend to be copious but dominated by few species, while specimen records from natural history collections and especially taxonomic literature tend to be fewer in number, but are often the only source of data on rare species. The Plazi approach gives free and persistent access to high quality data curated by taxonomic experts that might potentially help to identify and close knowledge gaps for some underrepresented groups.

**Table 2:**
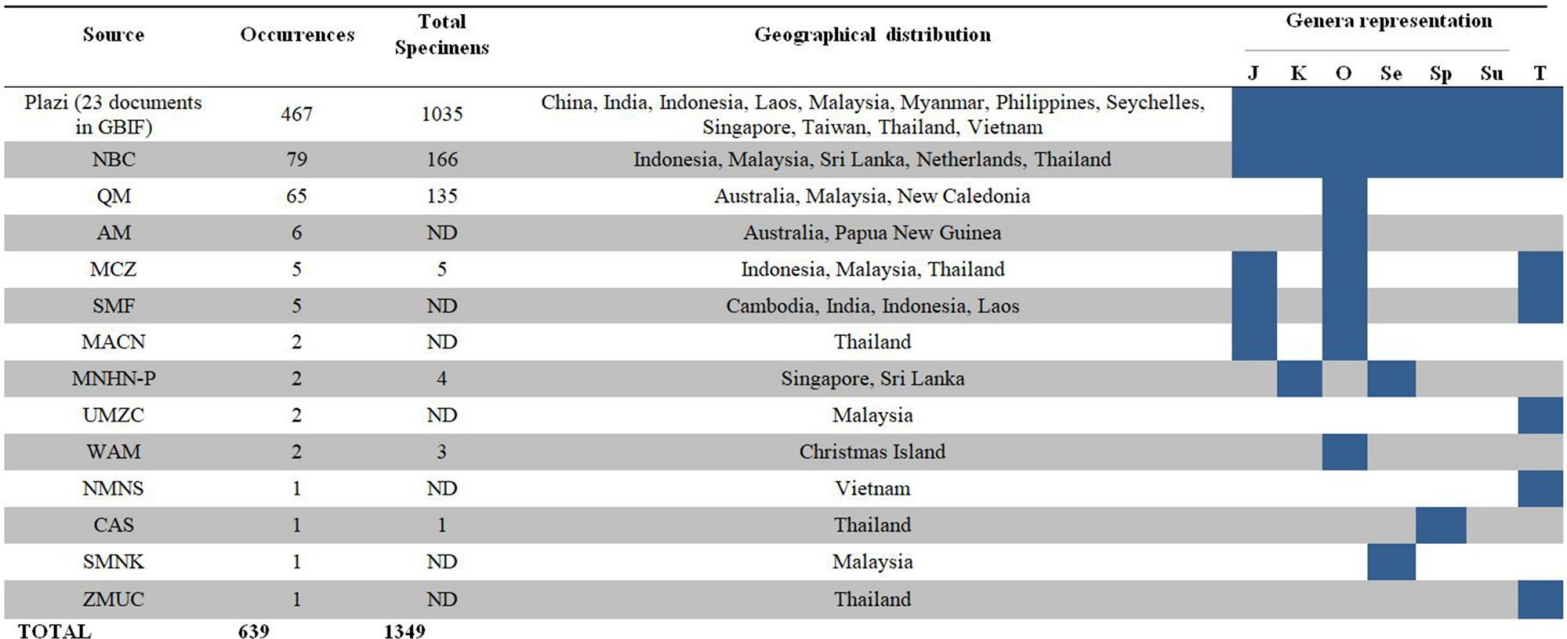
*Teutamus* group in GBIF per collection/database comparing number of occurrences, total of specimens, geographical distribution and taxonomic coverage.

Blue shaded squares indicate presence of each genus.

*J* *Jacaena*, *K* *Koppe*, *O* *Oedignatha*; *Se* *Sesieutes*, *Sp* *Sphingius*, *Su* *Sudharmia*, *T* *Teutamus*. Collection names: *AM* Australian Museum, Australia, *CAS* California academy of Sciences, USA; *MACN* Museo Argentino de Ciencias Naturales “Bernardino Rivadavia”, Argentina; *MCZ* Museum of Comparative Zoology, Harvard, USA; *MNHN–P* Muséum national d'Histoire naturelle-Paris, France; *NBC* Naturalis Biodiversity Center (formerly RMNH), The Netherlands; *NMNS* National Museum of Nature and Science, Japan; *QM* Queensland Museum, Australia; *SMF* Senckenberg Museum Frankfurt, Germany; *SMNK* Staatliches Museum für Naturkunde Karlsruhe, Germany; *UMZC* The University Museum of Zoology, Cambridge, UK; *WAM* West Australia Museum, Australia; *ZMUC* Zoological Museum, Natural History Museum, Denmark.

**Figure 4:**
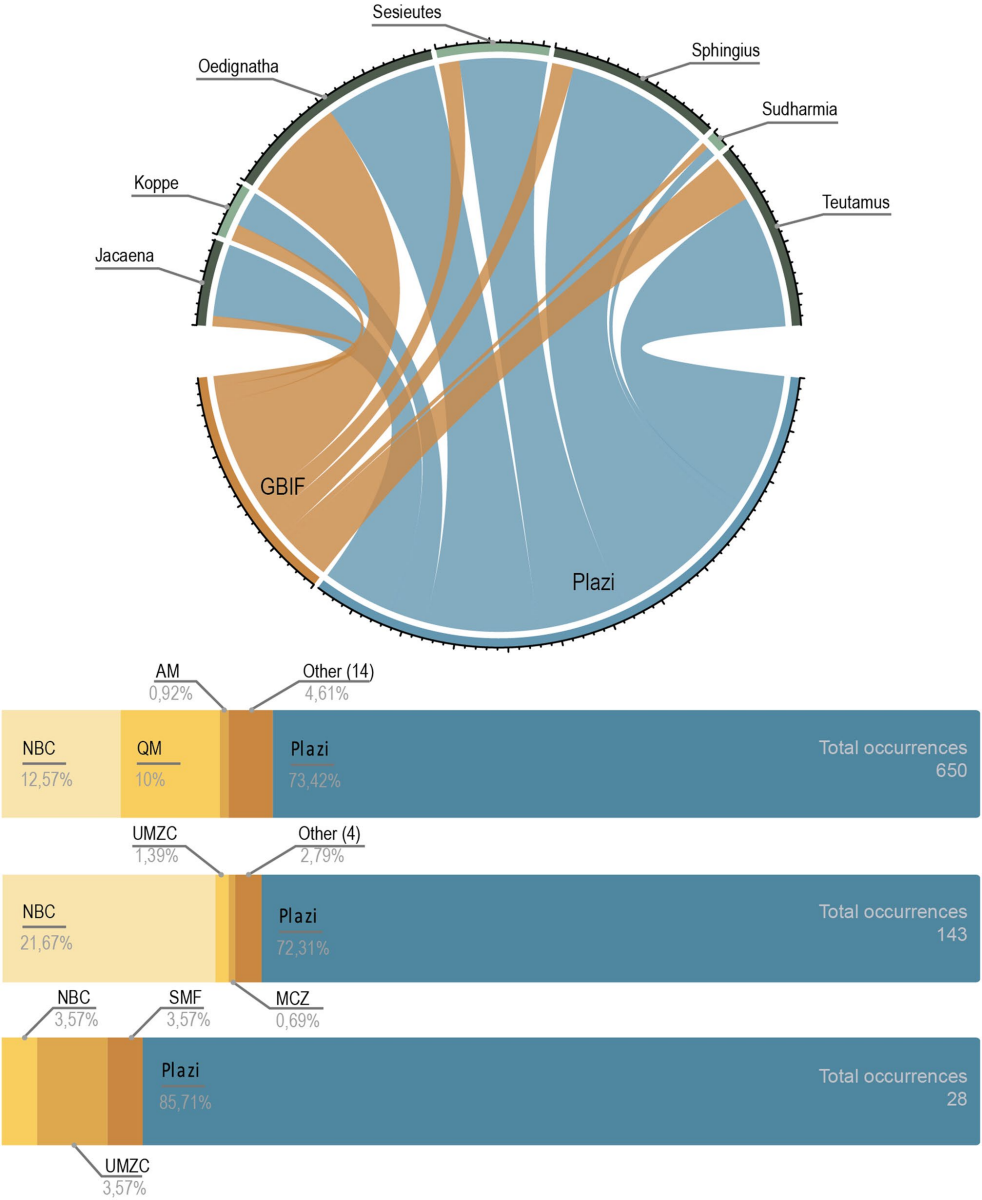
Proportion of occurrences of the *Teutamus* group in GBIF^65^. Color indicates data source: digitized collection data (brown shaded) and taxonomic literature mined data (blue). Circle: Proportion per data source for the whole Teutamus group and each TG genera. Generated in RStudio^28,68^. Bars: detail of proportions and total occurrences TG (top), genus Teutamus (middle), and Teutamus politus (middle). Note the high proportion of data contributed through our mark-up and integration using Plazi’s retrospective workflow). Collection abbreviations explained in Table 2.

Observation networks are some of the largest contributors to GBIF in terms of total records, but these tend to be quite limited in taxonomic focus and rarely include any but the most conspicuous and recognizable representatives of small bodied, high diversity groups like spiders. Here we emphasize the usefulness of the Plazi retrospective approach to close those gaps. Comparing a list of the currently valid species of the TG from the world spider catalog^27^, the Plazi approach contributed with records on 89 out of 137 species. By contrast, only 41 species of the TG were present in GBIF before our study. Our contributions to the knowledge of these spiders can be also observed in the number of occurrences in GBIF. Literature extracted data on the TG currently represents 470 occurrences in GBIF versus the 180 occurrences that were available from collection-based data, observation and iBOL combined. Our marked-up documents account for 72% of the occurrences of the TG and the genus *Teutamus*, and 85% of records of our target species, *Teutamus politus* (Fig. 4)*.* This gives evidence of the complementarity of these data sources and the importance of mobilizing and making publicly available all the specimen data contained in taxonomic literature.

It is worth noting that this complementarity can also mean that some records from literature and digitized collection data could be overlapping. However, ruling out these cases demands unambiguous collection numbers or specimen identifiers; or, in case this number is absent, comparing probable matches by collection date, locality, specimen count, and other data. For the *Teutamus* group, some records available in GBIF do have a unique collection number (e.g. *Teutamus politus* RMNH.ARA.15194). However, these identifiers are not always available (either in GBIF, on literature or on both) making difficult to reconcile data from different sources. Therefore setting unique identifiers and strengthening publication standards must be a top priority for the future^12,69–72^. This will help to generate usable and reliable datasets that can help to observe, study, and ultimately preserve biodiversity.

Structured, digitized specimen data extracted from taxonomic literature remains a small portion of the overall biodiversity data sphere, but it complements more mainstream data sources in important ways and has the potential to grow into a major source of data in its own right. Our study shows the importance of taxonomic literature records that, in combination with data from other sources, contributes to the most complete available assessment of spatial and temporal biodiversity pattern. Using this data for field work planning is but one possible application, but conservation risk assessment and species distribution modeling could be important in this context as well. The Plazi approach makes these data permanently available for others to re-use and add to in ways that we may or may not be able to currently imagine. Despite decades of ambitious and largely successful digitization efforts, much of the knowledge that biologists have accumulated about global biodiversity remains undigitized and unstructured, unqueryable, and difficult to access. The challenges presented by the global biodiversity crisis are daunting, and our best hope for addressing it begins with building a data infrastructure that faithfully represents the knowledge that generations of scientists have accumulated; specimen records from taxonomic literature are a key element in such an infrastructure.

## Methods

### Literature data extraction

We accessed all taxonomic literature of the family Liocranidae available in the World Spider Catalog^27^. We selected 55 publications that contained taxonomic treatments of the family Liocranidae^19–23,31–48,73–107^ (for full list, see Supplementary Table 1). We selected and processed all publications that provided taxonomic treatments with specimen data and usable geographical references. Publications written in a language other than English were not processed since OCR parsing, as implemented by the programs used here, has mostly been developed in this language. From the marked-up documents, 21 contained information on members of the TG and two on the species *T. politus*. We used the program GoldenGATE Imagine V.3 (GGI; https://plazi.org/resources/treatmentbank/goldengate-editor/) to semantically enhance PDF documents, allowing atomization and categorization of data. In some cases, ABBYY FineReader V. 11 was used first to extract and correct text from the PDF document using optical character recognition (ORC) and text editing functions. Once the PDF documents were marked and revised, we used GoldenGATE to upload the files to Plazi’s TreatmentBank^14^.

### Data analysis

We used Plazi Treatment Collection Statistics tool (https://tb.plazi.org/GgServer/srsStats) to download all the information relevant to our study in an excel spreadsheet to facilitate fine-grained management and analysis, largely following the approach described by Miller et al. (2015). We used these specimen based data to create profiles of the TG species allowing us to visualize where and when these taxa had been collected. Also, we used the GBIF occurrence search tool (https://www.gbif.org/occurrence/search) to look for records on our relevant TG taxa. The specific datasets we used can be found in the Data Accessibility section.

### Site selection

Literature data were used to design our field collection in a way that allowed us to optimize the collection of adult specimens of our target taxa in Southeast Asia (SEA). We explored the number of specimens of the TG reported per country, province and location whenever possible. We favored those locations with a higher representation of genera from the TG but also those where *T. politus* had been reported. Finally, we analyzed the total number of adult specimens collected per month for both the TG species and *T. politus* in order to increase the chances of finding adult spiders*.* Based on this, we decided to sample in three provinces in Thailand between July 16 and August 12, 2018.

### Sampling

Following the results of our literature analysis, we prioritized collections in national parks and protected areas. Precise geographical coordinates and specific habitat information was scarce or missing altogether in most taxonomic treatments. Therefore, we further divided each site in four different vegetation types (collecting sites details in the Supplementary Table 2) allowing us to cover a wide range of available habitats. We combined pitfall traps, Winkler extractors (for soil arthropods; www.entowinkler.at), and direct collecting targeting ground spiders. A mixture of propylene-glycol and ethanol was used in the pitfalls to avoid excessive evaporation and help with DNA preservation^108^; all specimens were collected and stored in 96% ethanol. All liocranid spiders were identified to species level. Juvenile spiders were assigned to a species only when they were at a pre-adult or late juvenile instar and adults were present in the same sample minimizing ambiguities.

## Supplementary information

Supplementary file1

## Data Availability

Extracted data is available from Plazi^14^ tb.plazi.org/GgServer/srsStats (refining search as needed) and GBIF^65,109,110^. A list of all the Plazi document UUID used in this study can be found in the Supplementary Table 1.
