# Supplementary material for: Mining data from legacy taxonomic literature and application for sampling spiders of the Teutamus group (Araneae; Liocranidae) in Southeast Asia
Source: Sci Rep. 2020 Sep 25;10:15787. doi: 10.1038/s41598-020-72549-8 (PMC7519673; doi:10.1038/s41598-020-72549-8)
Supplement: Supplementary file 1 — Supplementary file1 [file 41598_2020_72549_MOESM1_ESM.docx]

**Mining data from legacy taxonomic literature and application for sampling spiders of the *Teutamus* group (Araneae; Liocranidae) in Southeast Asia**

**Running title: Taxonomic legacy data extraction and field application**

**F. ANDRES RIVERA-QUIROZ***^1, 2^, **BOOPPA PETCHARAD**^3^ and **JEREMY A. MILLER**^1^

^1^ Department of Terrestrial Zoology, Understanding Evolution group, Naturalis Biodiversity Center, Darwinweg 2, 2333CR Leiden, The Netherlands

E-mail: rivera.andres37@gmail.com; jeremy.miller@naturalis.nl.

^2^ Institute of Biology Leiden (IBL), Leiden University, Sylviusweg 72, 2333BE Leiden, The Netherlands

^3^ Faculty of Science and Technology, Thammasat University, Rangsit, Pathum Thani, 12121 Thailand.

^4^ Plazi, Zinggstrasse 16, CH 3007, Bern, Switzerland

*Corresponding author

E-mail: [andres.riveraquiroz@naturalis.nl](mailto:andres.riveraquiroz@naturalis.nl) (AR)

**Supplementary Figure 1.** Visual summary of the data extraction process for *Teutamus politus* treatment from Dankittipakul, Tavano, and Singtripop (2012). 1- Taxonomic document in PDF format downloaded from the World Spider Catalog <https://wsc.nmbe.ch/species/7486>. 2- Conversion to XML format using Golden Gate Imagine. Each color in the figure text represents a semantic tag in the extraction process (Sautter, Böhm, and Agosti 2007). 3- Extracted treatment as displayed in Plazi <http://tb.plazi.org/GgServer/html/03A6879FA845FFA9E5BCFB740217658D>. To the left, whole treatment text, illustrations and link to the original source, to the right charts and maps based on the specimen data. 4- Specimen data and taxonomic treatment text displayed in GBIF <https://www.gbif.org/species/130509488>.


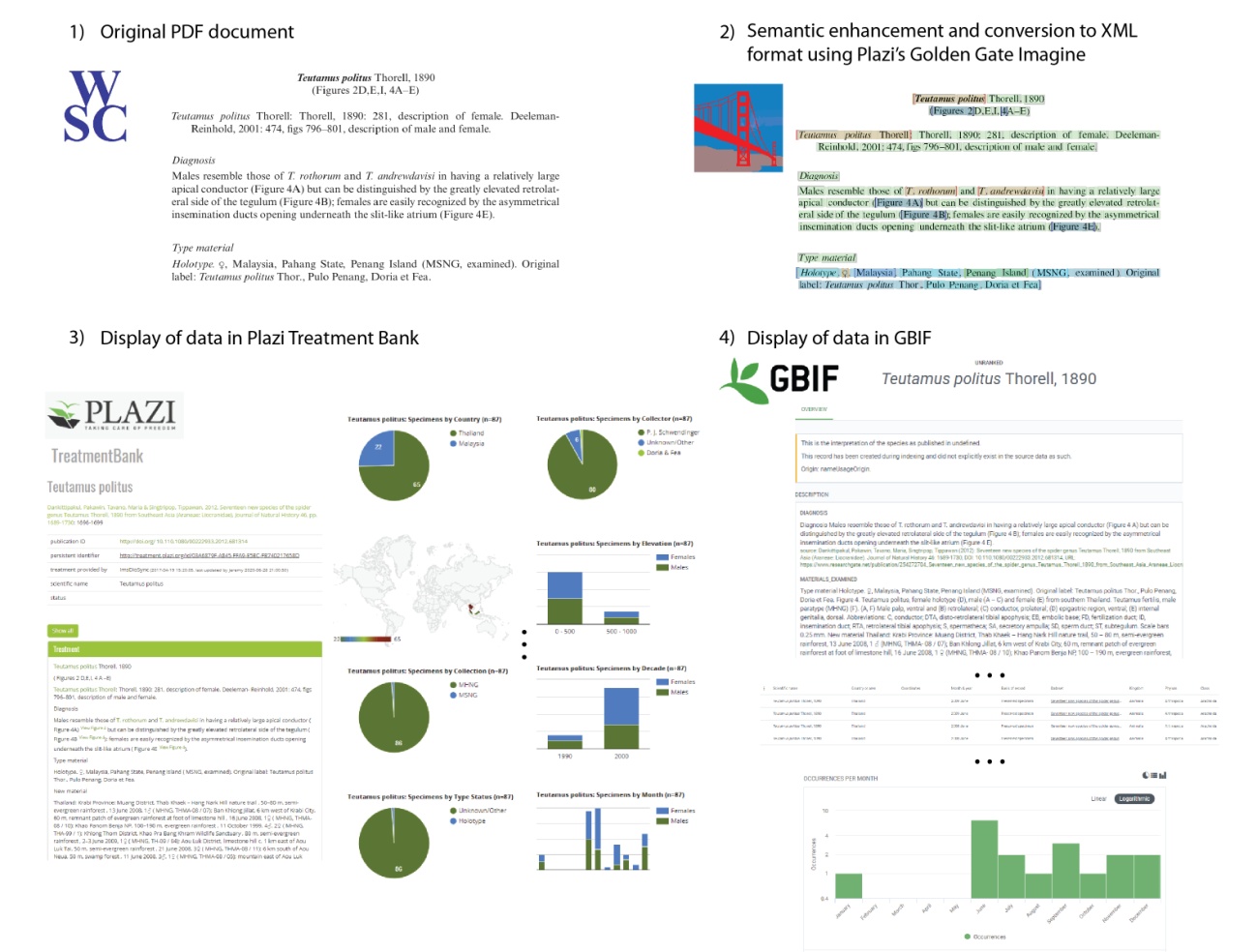


**Supplementary Figure 2.** Proportion of GBIF records per taxonomic groups. Left circle represent the whole GBIF database. Right circle represent spiders and other arachnids detailing the proportion of the best represented spider families and the Liocranidae (in red).


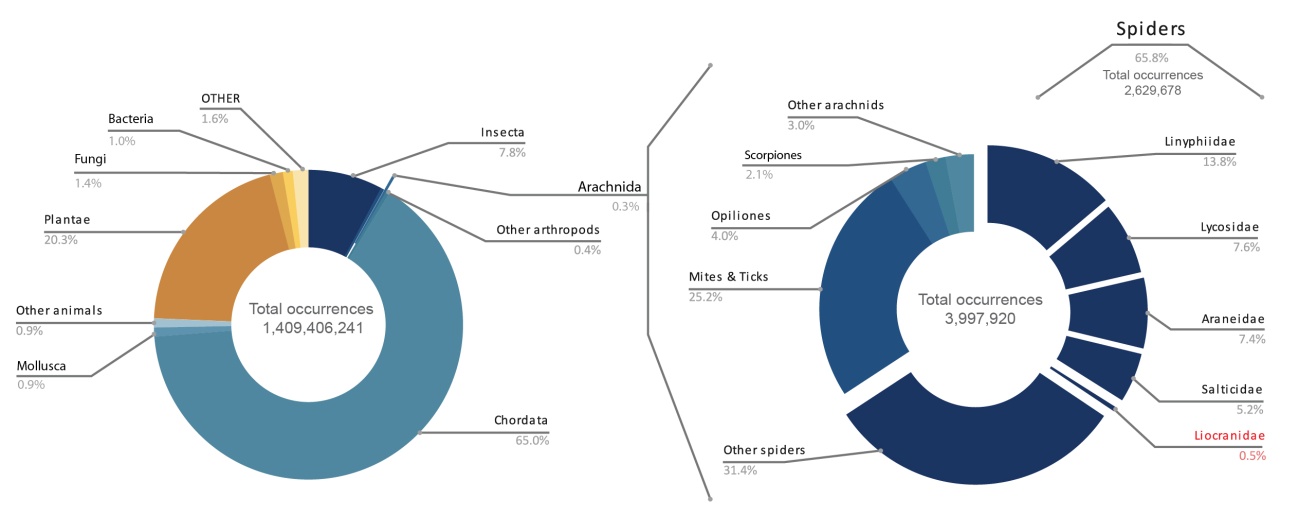


**Supplementary Table 1.** Complete list of processed publications of the family Liocranidae. Plazi UUID code (a unique persistent identifier given to documents), number of liocranid genera and species treated, and specimens listed per study. The UUID can be added to the prefix this prefix “http://tb.plazi.org/GgServer/summary/” to access the index of linked treatments for that source. A list of the references used in this table can be found at the end of this supplementary document.

| **Study** | **Article UUID** | **Non TG** | | | **TG** | | | **Total specimens** |
| --- | --- | --- | --- | --- | --- | --- | --- | --- |
|  |  | **Genera** | **Species** | **Specimens** | **Genera** | **Species** | **Specimens** |  |
| Barrion and Litsinger 1995 | D6116953413AA950FFBB4926252AFFE1 | - | - | - | 3 | 3 | 8 | 8 |
| Bastawade 2006 | E370626DFFC6FFDCFFB4FFFFFFC3E666 | - | - | - | 1 | 1 | 12 | 12 |
| Bastawade 2002 | FFCC8B0CFFA3FF80A43F537CFFC6F93F | - | - | - | 1 | 1 | 3 | 3 |
| Bennett, Copley, and Copley 2013 | 8F7EFFBB8872FFB96C32B8600B71A62C | 1 | 1 | 114 | - | - | - | 114 |
| Biswas and Raychaudhuri 2000 | FFEEFFB22C17FFEEFF9E8F29FFCBFFD8 | - | - | - | 1 | 1 | 14 | 14 |
| Biswas and Roy 2008 | FFF2D503FFD8154B7903FF201A02FF92 | 1 | 1 | 6 | - | - | - | 6 |
| Biswas and Biswas 1992 | 06598512FFD6FFEFFF80FFD3F611FFF8 | - | - | - | 2 | 2 | 9 | 9 |
| Biswas and Majumder 1995 | 8F48FF87FF9A9839B635FFD6FF85DD64 | - | - | - | 1 | 1 | 1 | 1 |
| Bosmans and van Keer 2012 | DE294F64CD29FFFCCA795F1BFF93FFAA | 1 | 1 | 2 | - | - | - | 2 |
| Bosmans 1999 | FFAAFF87FFCDFFD9FF87461AFFA12804 | 4 | 7 | 339 | - | - | - | 339 |
| Bosselaers 2009 | FF805162FFD1F162FFF3F327FF8FD505 | 4 | 5 | 109 | - | - | - | 109 |
| Bosselaers 2012 | FF9CFFB9FB60691AFFEAFF9C85151220 | 1 | 1 | 1 | - | - | - | 1 |
| Bosselaers et al. 2010 | 7F15FF9E4C7DFF817E28ED77920EEC4D | 1 | 1 | 2 | - | - | - | 2 |
| Candek et al. 2013 | 622F99677618FFF81D122429FFE4FF91 | 2 | 2 | 6 | - | - | - | 6 |
| Chen and Huang 2009 | FFEEFFF5C520246D3960E4674C4EE517 | - | - | - | 1 | 1 | 24 | 24 |
| Crespo et al. 2018 | FFA7F823FFB5FFF26305FFECFFC5B633 | 4 | 9 | 70 | - | - | - | 70 |
| Danilov 1998 | 733D3830FFB3FFFFFFF4FFC8FFAFFF8F | 1 | 3 | 21 | - | - | - | 21 |
| Dankittipakul and Deeleman-Reinhold 2012 | FA379502FFBEFFB0FFF8FFC06931FFFB | - | - | - | 1 | 1 | 9 | 9 |
| Dankittipakul and Deeleman-Reinhold 2013 | FFF7FFD5FFEF5F40FF83FFDAFFBE5660 | - | - | - | 1 | 9 | 54 | 54 |
| Dankittipakul, Tavano, and Singtripop 2011 | 566E5A667339121BA634FFDC3833FF8F | - | - | - | 1 | 13 | 79 | 79 |
| Dankittipakul, Tavano, and Singtripop 2012 | FF9FFFE7A842FFA3E422FFF90169654C | - | - | - | 1 | 18 | 205 | 205 |
| Dankittipakul, Tavano, and Singtripop 2013 | 10136F22FFB5FF85AE6DD059FFD39809 | - | - | - | 1 | 9 | 43 | 43 |
| Deeleman-Reinhold 2001 | FF8A860AC93EFFE765528965DD50FFD6; FF8FE1734262FFA4FF8A4255DE29FF94; FFC9FFC63A1BFFDDFFD5FFC6DB37324B | 1 | 1 | 6 | 7 | 38 | 564 | 570 |
| Deltshev et al. 2013 | FFD9FFE35975FFA9FFF1FFC1FFCDFF82 | 2 | 2 | 2 | - | - | - | 2 |
| Deltshev and Wang 2016 | FFB8FFF5C324E437FFF7FF910D10FC7A | 1 | 1 | 4 | - | - | - | 4 |
| Elverici, Özkütük, and Kunt 2013 | FFA7C048B65EFFF5AE774A45FF9FF24A | 2 | 2 | 25 | - | - | - | 25 |
| Esyunin and Kazantsev 2007 | 5141AC78642A420DDF7CFFDFFFFB3311 | 1 | 1 | 4 | - | - | - | 4 |
| Felton, Judd, and Merrett 2004 | FFCCFF95D602FF82FF84FF82FFF1E808 | 1 | 1 | 6 | - | - | - | 6 |
| Fu, Zhang, and Zhu 2009 | 4C1488709C14A741FF9CFFE8FF98FFB9 | 1 | 1 | 17 | - | - | - | 17 |
| Hayashi 1992 | FF9896040E0FFF90512CFFD1FFE9FFDC | 1 | 2 | 4 | - | - | - | 4 |
| Jäger 2007 | 2E4DFFC7FF823B04FF8434627515FFF5 | - | - | - | 1 | 1 | 1 | 1 |
| Bosselaers and Jocqué 2013 | 9641FFF01026FF93FFC1CF4E123CD00A | 1 | 7 | 227 | - | - | - | 227 |
| Jonsson 2005 | FFB49220FFB2FF91FFD2FFA9C165DB4A | 1 | 1 | 7 | - | - | - | 7 |
| Marusik and Koponen 2000 | BE33BE057603DB13FFDE024BCB37C220 | 1 | 1 | 1 | - | - | - | 1 |
| Marusik, Omelko, and Koponen 2016 | 3F632B49FFD3296A0D5CA5685D01FFB9 | 1 | 2 | 3 | - | - | - | 3 |
| Marusik, Zheng, and Li 2008 | F43D9C12004C613DA434AC5324270C62 | 1 | 2 | 16 | - | - | - | 16 |
| Namkung 1989 | FFBDFFD5063A094CFF937B5D13709146 | 1 | 1 | 3 | - | - | - | 3 |
| Ono 2009 | 3513FF9301781725463FFF92FFA2FF89 | - | - | - | 1 | 1 | 1 | 1 |
| Platnick and Di Franco 1992 | 4C59FFB0FFE19E6FFFB7FFD0FFEDFF8B | 1 | 6 | 19 | - | - | - | 19 |
| Reboleira et al. 2012 | D40BFFF1FF9DFFC7FFB3FFD2FFE3B822 | 2 | 4 | 40 | - | - | - | 40 |
| Reddy and Patel 1993 | AF35FFE0FF850E68FFA5145FFFE6FFEB | - | - | - | 1 | 1 | 3 | 3 |
| Ribera and de Mas 2015 | 6864FFE79E0A1704FFFCFF8EFFBAF854 | 1 | 9 | 19 | - | - | - | 19 |
| Bosmans 2011 | FFE1FF97FFF5FFBFFFA5FFC935544357 | 2 | 2 | 11 | - | - | - | 11 |
| Saaristo 2002 | FF809205FFF21921FFC75A4699324D69 | - | - | - | 1 | 2 | 236 | 236 |
| Sankaran et al. 2017 | 8618FF9AFF914734FFD0FFCF3A19FF86 | 1 | 1 | 17 | - | - | - | 17 |
| Seo 2011 | FF8BB520FFA9743EFFF97A74FFBAF37E | 2 | 3 | 9 | - | - | - | 9 |
| Seyyar et al. 2016 | FFA1FFC04B7C6416FFAEE21F200EFFDC | 1 | 1 | 3 | - | - | - | 3 |
| Tso et al. 2005 | BD41FFEDE627A13FB559E159D628093C | - | - | - | 2 | 2 | 2 | 2 |
| Ubick and Platnick 1991 | FFB7AC78821CFFC2FFC2FFD7B54DAD40 | 1 | 1 | 41 | - | - | - | 41 |
| Ubick and Vetter 2005 | F117A4286D45FFE7233FFFA2FF906E1E | 1 | 1 | 109 | - | - | - | 109 |
| Vetter 2001 | FF9BFFDBFFB54A58AC05FFCDFFD2FFAB | 1 | 2 | 320 | - | - | - | 320 |
| Warui and Jocqué 2002 | FFDE4808FFABFFDCFFEDFFAFF36A181D | 1 | 2 | 28 | - | - | - | 28 |
| Wunderlich 2011 | FFC1DD35277DCD7FBE6DFE7EFFCAFFE0 | 2 | 3 | 13 | - | - | - | 13 |
| Zapata and Ramírez 2010 | 5924FF98422FFFB9FF823E088233FFC8 | 1 | 1 | 1 | - | - | - | 1 |
| Zhang and Fu 2010 | FF806164FFE5FFDF2F48FF90FFF6D054 | - | - | - | 1 | 1 | 15 | 15 |
| Zhang, Fu, and Zhu 2009 | FF8A9941EF368C07FFC8FFC4FFB1A240 | - | - | - | 1 | 4 | 23 | 23 |
| Zhao and Peng 2013 | 8163FFFD6B76FFA8FFA5FFEE764AFFB6 | 1 | 1 | 3 | 2 | 2 | 3 | 6 |
| Zonstein, Marusik, and Omelko 2015 | FFA288738C19FFC1FFA7FFD054431A6B | 1 | 1 | 8 | - | - | - | 8 |
|  |  | **55** | **94** | **1636** | **32** | **112** | **1309** | **2945** |

**Supplementary Table 2**. Detail of our sampling sites in Thailand.

| Province | Site details | Geographic Coordinates and elevation | Date |
| --- | --- | --- | --- |
| Chiang Mai | Pha Daeng NP. Riparian tropical forest. | 19º37.768'N 98º57.257'E, 560m. | 16-19 July 2018. |
|  | Pha Daeng NP. Bamboo forest. | 19º37.668'N 98º57.131'E, 573m. | 16-19 July 2018. |
|  | Pha Daeng NP. Mixed Teak forest. | 19º34.320'N 98º57.340'E, 474m. | 16-19 July 2018. |
|  | Pha Daeng NP. Dipterocarpus forest. | 19º36.132'N 98º56.980'E, 571m. | 17-19 July 2018. |
|  | Doi Inthanon NP. Cloud forest. | 18º35.268'N 98º29.240'E, 2572m. | 21-24 July 2018. |
|  | Doi Inthanon NP. Montane evergreen forest. | 18º30.454'N 98º30.584'E, 1605m. | 21-24 July 2018. |
|  | Doi Inthanon NP. Mixed pine forest. | 18º32.606'N 98º34.479'E, 995m. | 21-24 July 2018. |
|  | Doi Inthanon NP. Mixed oak-pine tropical forest. | 18º32.436'N 98º31.858'E, 1279m. | 21-24 July 2018. |
|  | Doi Suthep NP. Montane evergreen forest with pine. | 18º48.502'N 98º53.528'E, 1409m. | 24-28 July 2018. |
|  | Doi Suthep NP. Mixed oak tropical forest. | 18º48.164'N 98º54.081'E, 1300m. | 24-28 July 2018. |
|  | Doi Suthep NP. Mixed bamboo tropical forest. | 18º49.045'N 98º55.296'E, 802m. | 25-28 July 2018. |
|  | Doi Suthep NP. Dipterocarpus forest. | 18º48.780'N 98º55.928'E, 643m. | 25-28 July 2018. |
| Phuket | Ton Sai Waterfall. Mixed bamboo tropical forest. | 8º1.673'N 98º22.019'E, 144m. | 29 July - 2 August 2018. |
|  | Ton Sai Waterfall. Mixed Kerriodoxa elegans tropical forest. | 8º1.816'N 98º22.375'E, 215m. | 29 July - 2 August 2018. |
|  | Bang Pae Waterfall. Mixed bamboo tropical forest. | 8º2.310'N 98º23.407'E, 135m. | 30 July - 3 August 2018. |
|  | Bang Pae Waterfall. Mixed tropical forest. | 8º2.353'N 98º23.365'E, 173m. | 31 July - 4 August 2018. |
|  | Siray Island. Mixed tropical forest. | 7º53.355'N 98º26.083'E, 132m. | 2-6 August 2018. |
|  | Siray Island. Rubber plantation. | 7º53.384'N 98º26.102'E, 104m. | 2-6 August 2018. |
|  | Siray Island. Mixed tropical forest near banana plantation. | 7º53.169'N 98º26.108'E, 88m. | 3-6 August 2018. |
|  | Siray Island. Mixed tropical forest near rubber plantation. | 7º53.409'N 98º26.067'E, 117m. | 4 August 2018. |
| Krabi | Community Forest near Than Bok Khorani NP. Mixed tropical forest. | 8º29.536'N 98º44.353'E, 93m. | 7-12 August 2018. |
|  | Community Forest near Than Bok Khorani NP. Mixed bamboo tropical forest. | 8º29.572'N 98º44.367'E, 85m. | 8-12 August 2018. |
|  | Community Forest near Than Bok Khorani NP. Mixed young tropical forest. | 8º29.655'N 98º44.001'E, 60m. | 9-12 August 2018. |
|  | Community Forest near Than Bok Khorani NP. Oil palm plantation. | 8º29.592'N 98º43.907'E, 56m. | 9 August 2018. |

**Reference list**

Barrion, AT, & JA Litsinger. (1995). Family Clubionidae Wagner- Genera Alaeho, Castianeira, Agroeca, Phrurolithus & Scotinella. In Riceland Spiders of South and Southeast Asia, 170–80. DOI: 10.5281/zenodo.897849.

Bastawade, DB. (2002). Three New Species from the Spider Families Amaurobiidae, Thomisidae and Salticidae (Araneae: Arachnida) from India. Journal of the Bombay Natural History Society 99:274–81.

———. (2006). Replacement Name for Amaurobius Indicus Bastawade and Its Transfer to Family Corinnidae (Arachnida: Araneae). Zoo’s Print Journal 21:2307.

Bennett, R, C Copley, & D Copley. (2013). Apostenus Ducati (Araneae: Liocranidae) Sp. Nov.: A Second Nearctic Species in the Genus. Zootaxa 3647 (1):63–74. 10.11646/zootaxa.3647.1.3.

Biswas, B, & K Biswas. (1992). Araneae: Spiders. In State Fauna Series 3: Fauna of West Bengal 3, 357–500.

Biswas, B, & SC Majumder. (1995). Araneae: Spider. In Fauna of Meghalaya, State Fauna Series. Zoological Survey of India Kolkata, 93–128.

Biswas, V, & D Raychaudhuri. (2000). Sac Spiders of Bangladesh-II: Genera Castianeira Keyserling, Sphingius Thorell and Trachelas Koch (Araneae: Clubionidae). Records of the Zoological Survey of India 98:131–39.

Biswas, V, & R Roy. (2008). Description of Six New Species of Spiders of the Genera Lathys (Family: Dictynidae), Marpissa (Family: Salticidae), Misumenoides (Family: Thomisidae), Agroeca (Family: Clubionidae), Gnaphosa (Family: Gnaphosidae) and Flanona (Family: Lycosidae) - F. Records of the Zoological Survey of India 108:43–57.

Bosmans, R. (1999). The Genera Agroeca, Agraecina, Apostenus and Scotina in the Maghreb Countries (Araneae: Liocranidae). Bulletin de l’ Institut Royal de Sciences Naturelles de Belgique 69:25–34.

———. (2011). On Some New or Rare Spider Species from Lesbos, Greece (Araneae: Agelenidae, Amaurobiidae, Corinnidae, Gnaphosidae, Liocranidae). Arachnologische Mitteilungen, no. 40:15–22. 10.5431/aramit4003.

Bosmans, R, & J van Keer. (2012). On the Spider Species Described by L. Koch in 1882 from the Balearic Islands (Araneae). Arachnologische Mitteilungen 43:5–16. doi:10.5431/aramit4306.

Bosselaers, J. (2009). Studies in Liocranidae (Araneae): Redescriptions and Transfers in Apostenus Westring and Brachyanillus Simon, as Well as Description of a New Genus. Zootaxa, no. 2141:37–55.

———. (2012). Two Interesting New Ground Spiders (Araneae) from the Canary Islands and Greece. Serket 13:83–90.

Bosselaers, J, M Dierick, V Cnudde, B Masschaele, L Van Hoorebeke, & P Jacobs. (2010). High-Resolution X-Ray Computed Tomography of an Extant New Donuea (Araneae: Liocranidae) Species in Madagascan Copal. Zootaxa, no. 2427:25–35.

Bosselaers, J, & R Jocqué. (2013). Studies in Liocranidae (Araneae): A New Afrotropical Genus Featuring a Synapomorphy for the Cybaeodinae. European Journal of Taxonomy 40:1–49.

Candek, K, M Gregorič, R Kostanjšek, H Frick, C Kropf, & M Kuntner. (2013). Targeting a Portion of Central European Spider Diversity for Permanent Preservation. Biodiversity Data Journal 1:e980. 10.3897/BDJ.1.e980. eCollection 2013.

Chen, SH, & WJ Huang. (2009). A Newly Recorded Spider Oedignatha Platnicki Song et Zhu 1998 from Taiwan, with Description of the Female (Araneae, Corinnidae). BioFormosa 44:31–36.

Crespo, LC, M Domènech, A Enguídanos, J Malumbres-Olarte, P Cardoso, J Moya-Laraño, C Frías-López, et al. (2018). A DNA Barcode-Assisted Annotated Checklist of the Spider (Arachnida, Araneae) Communities Associated to White Oak Woodlands in Spanish National Parks. Biodiversity Data Journal 6:e29443. doi:10.3897/BDJ.6.e29443.

Danilov, SN. (1998). The Spider Family Liocranidae in Siberia and Far East (Aranei). Arthropoda Selecta 7:313–17.

Dankittipakul, P, & C Deeleman-Reinhold. (2012). A New Spider Species of the Genus Sudharmia from Sumatra, Indonesia (Araneae, Liocranidae). Dongwuxue Yanjiu 33 (2):187–90. 10.3724/SP.J.1141.2012.02187.

———. (2013). Delimitation of the Spider Genus Sesieutes Simon, 1897, with Descriptions of Five New Species from South East Asia (Araneae: Corinnidae). Journal of Natural History 47 (3–4):167–95. 10.1080/00222933.2012.742165.

Dankittipakul, P, M Tavano, & T Singtripop. (2011). Neotype Designation for Sphingius Thecatus Thorell 1890 Synonymies New Records and Descriptions of Six New Species from Southeast Asia(Araneae Liocranidae). Zootaxa, no. 3066:1–20.

———. (2012). Seventeen New Species of the Spider Genus Teutamus Thorell, 1890 from Southeast Asia (Araneae: Liocranidae). Journal of Natural History 46 (27–28):1689–1730. 10.1080/00222933.2012.681314.

———. (2013). Revision of the Spider Genus Jacaena Thorell, 1897, with Descriptions of Four New Species from Thailand (Araneae: Corinnidae). Journal of Natural History 47 (23–24):1539–67. 10.1080/00222933.2012.763059.

Deeleman-Reinhold, C. (2001). Forest Spiders of South East Asia: With a Revision of the Sac and Ground Spiders (Araneae: Clubionidae, Corinnidae, Liocranidae, Gnaphosidae, Prodidomidae and Trochanterriidae). Leiden : Brill: 591.

Deltshev, C, M Komnenov, G Blagoev, T Georgiev, S Lazarov, E Stojkoska, & M Naumova. (2013). Faunistic Diversity of Spiders (Araneae) in Galichitsa Mountain (FYR Macedonia). Biodiversity Data Journal 1:e977. 10.3897/BDJ.1.e977.

Deltshev, C, & C Wang. (2016). A New Agraecina Spider Species from the Balkan Peninsula (FYR Macedonia) (Araneae: Liocranidae). Zootaxa 4117 (1):135–40. 10.11646/zootaxa.4117.1.8.

Elverici, M, RS Özkütük, & KB Kunt. (2013). Two New Liocranid Species Records from Turkey (Araneae: Liocranidae). Munis Entomology and Zoology 1 (1):305–8.

Esyunin, SL, & DK Kazantsev. (2007). On the Spider (Aranei) Fauna of the Pechoro-Ilychskiy Reserve (North Urals), with the Description of a New Agroeca Species (Liocranidae). Arthropoda Selecta 16:245–50.

Felton, C, S Judd, & P Merrett. (2004). Agroeca Dentigera Kulczynski, 1913, a Liocranid Spider New to Britain (Araneae, Liocranidae). Bulletin of the British Arachnological Society 13 (3):90–92.

Fu, JY, F Zhang, & MS Zhu. (2009). Redescription of a Little-Known Spider Species, Mesiotelus Lubricus (Simon, 1880) (Aranei: Liocranidae) from China. Arthropoda Selecta 17:169–73.

Hayashi, T. (1992). Three Species of the Genus Agroeca (Araneae: Clubionidae) from Japan, Including a New Species. Acta Arachnologica 41:133–37.

Jäger, P. (2007). Spiders from Laos with Descriptions of New Species (Arachnida: Araneae). Acta Arachnologica 56:29–58.

Jonsson, LJ. (2005). Agroeca Dentigera and Entelecara Omissa (Araneae: Liocranidae, Linyphiidae), Found in Sweden. Arachnologische Mitteilungen, no. 29:49–52.

Marusik, YM, & S Koponen. (2000). New Data on Spiders (Aranei) from the Maritime Province, Russian Far East. Arthropoda Selecta 9:55–68.

Marusik, YM, MM Omelko, & S Koponen. (2016). Rare and New for the Fauna of the Russian Far East Spiders (Aranei). Far Eastern Entomologist 317:1–15.

Marusik, YM, G Zheng, & S Li. (2008). A Review of the Genus Paratus Simon (Araneae, Dionycha). Zootaxa 1965 (1965):50–60.

Namkung, J. (1989). A New Species of the Genus Agroeca (Araneae: Clubionidae) from Korea. Korean Arachnology 5:23–27.

Ono, H. (2009). Three New Spiders of the Family Clubionidae, Liocranidae and Gnaphosidae (Arachnida, Araneae) from Vietnam. Bulletin of the National Museum of Nature and Science Tokyo 35:1–8.

Platnick, NI, & F Di Franco. (1992). On the Relationship of the Spider Genus Cybaeodes (Araneae, Dionycha). American Museum Novitates, no. 3053:9.

Reboleira, AS, AJ Pérez, H López, N Macías-Hernández, S de la Cruz, & P Oromí. (2012). Catalogue of the Type Material in the Entomological Collection of the University of La Laguna (Canary Islands, Spain). I. Arachnida. Zootaxa 3556:61–79.

Reddy, TS, & BH Patel. (1993). Two New Species Of The Genus Oedignatha Thorell (Araneae: Clubionidae) From Coastal Andhra Pradesh, India. Entomon 18:47–51.

Ribera, C, & E de Mas. (2015). Description of Three New Troglobiontic Species of Cybaeodes (Araneae, Liocranidae) Endemic to the Iberian Peninsula. Zootaxa 3957 (3):313–23. 10.11646/zootaxa.3957.3.4.

Saaristo, MI. (2002). New Species and Interesting New Records of Spiders from Seychelles (Arachnida, Araneaea). Phelsuma 10:1–32.

Sankaran, PM, JJ Malamel, MM Joseph, & PA Sebastian. (2017). A New Species of Paratus Simon, 1898 (Araneae: Liocranidae, Paratinae) from India. Zootaxa 4286:139–44. doi:10.11646/zootaxa.4286.1.12.

Sautter, G, K Böhm, & D Agosti. (2007). Semi-Automated XML Markup of Biosystematic Legacy Literature with the GoldenGATE Editor. *Pacific Symposium on Biocomputing. Pacific Symposium on Biocomputing* 402 (11):391–402.

Seo, BK. (2011). Description of Three Liocranid Spider Species from Korea (Araneae: Liocranidae). Entomological Research 41 (3):98–102. 10.1111/j.1748-5967.2011.00326.x.

Seyyar, O, A Oba, H Demir, & T Turkes. (2016). Arabelia Bosselaers, 2009 and Arabelia Pheidoleicomes Bosselaers, 2009 (Araneae: Liocranidae) Are New Records for the Turkish Spider Fauna. Serket 15 (1):30–32.

Tso, I, MS Zhu, J Zhang, & F Zhang. (2005). Two New and One Newly Recorded Species of Corinnidae and Liocranidae from Taiwan (Arachnida: Araneae). Acta Arachnologica 54:45–49.

Ubick, D, & NI Platnick. (1991). On Hesperocranum, A New Spider Genus from Western North America (Araneae, Liocranidae). American Museum Novitates, 1–12.

Ubick, D, & RS Vetter. (2005). A New Species of Apostenus From California, With Notes on the Genus (Araneae, Liocranidae). Journal of Arachnology 33 (1):63–75. 10.1636/H03-24.

Vetter, RS. (2001). Revision of the Spider Genus Neoanagraphis (Araneae,Liocranidae). Journal of Arachnology 29 (1):1–10. 10.1636/0161-8202(2001)029[0001:ROTSGN]2.0.CO;2.

Warui, C, & R Jocqué. (2002). The First Gallieniellidae (Araneae) from Eastern Africa. Journal of Arachnology 30 (2):307–15. 10.1636/0161-8202(2002)030[0307:TFGAFE]2.0.CO;2.

Wunderlich, J. (2011). On European Spiders of the Nominal Families Liocranidae, Miturgidae and Zoridae (Araneae), with Descriptions of New Taxa. Beiträge Zur Araneologie 6:108–20.

Zapata, L V., & MJ Ramírez. (2010). A New Species of the Genus Paratus Simon (Araneae: Liocranidae) from Thailand. Zootaxa, no. 2418:65–68.

Zhang, F, & JY Fu. (2010). First Report of the Genus Sesieutes Simon (Araneae: Liocranidae) from China, with Description of One New Species. Entomological News 121 (1):69–74. 10.3157/021.121.0114.

Zhang, F, JY Fu, & MS Zhu. (2009). Spiders of the Genus Sphingius (Araneae: Liocranidae) from China, with Description of Two New Species. Zootaxa, no. 2298:31–44.

Zhao, Y, & XJ Peng. (2013). Three New Species of Spiders of the Family Liocranidae (Arachnida: Araneae) from China. Oriental Insects 47 (2–3):176–83. 10.1080/00305316.2013.811021.

Zonstein, SL, YM Marusik, & M Omelko. (2015). A Survey of Spider Taxa New to Israel (Arachnida: Araneae). Zoology in the Middle East 61 (4):372–85. 10.1080/09397140.2015.1095525.
